# Le Coup de Poignard Rachidien: A Historical Perspective

**DOI:** 10.7759/cureus.4175

**Published:** 2019-03-04

**Authors:** Nidal B Omar, Joseph Miller, Mohammadali M Shoja, Mark R Harrigan, R. Shane Tubbs

**Affiliations:** 1 Neurosurgery, University of Alabama at Birmingham, Birmingham, USA; 2 Surgery, University of Texas Medical Branch, Galveston, USA; 3 Neurosurgery, Seattle Science Foundation, Seattle, USA

**Keywords:** spinal, subarachnoid, hemorrhage, michon

## Abstract

A spinal subarachnoid hemorrhage (SAH) is uncommon. One of the earliest detailed analyses of a spinal SAH was in 1928 by the French physician Paul Michon, who coined the term “le coup de poignard rachidien” to describe the pathognomonic, intense spinal pain experienced by patients with spinal SAH, equating it to being stabbed by a dagger. Michon sub-classified spinal SAH into the upper and lower forms, pointing out that the stabbing spinal pain is more characteristic of SAH in the cervical and thoracic regions and especially in the interscapular region. Translation and subsequent analysis of Michon’s original French paper published in La Presse Medicale in 1928 shed light on two cases in which patients presented with le coup de poignard rachidien and signs of spinal cord dysfunction but little, if any, intracranial symptoms. The patients both showed symptomatic relief following therapeutic lumbar puncture. Later, authors have questioned the notion that intense spinal or interscapular pain is mandatory in the diagnosis of spinal SAH and have additionally provided evidence contrary to Michon’s assertion that intracranial symptoms, if any, occur later in the progression of spinal SAH and are largely insignificant.

## Introduction and background

A spinal subarachnoid hemorrhage (SAH) is a rare condition. It is estimated that less than 1% of all cases of SAH are of spinal origin [1]. Furthermore, spinal SAH often presents a diagnostic challenge by producing symptoms that overlap with those of intracranial SAH [2]. One of the earliest works providing an in-depth analysis of this condition was by the French physician Paul Michon, who in 1928 published a paper titled Le coup de poignard rachidien: symptome initial de certaines hemorragies sous-arachnoidiennes. Although cases of spinal SAH had been described prior to Michon’s paper, and he cited many such works, he was the first to emphasize the acute and intense spinal pain, described as being “stabbed in the back by a dagger,” as part of the diagnostic criteria for certain types of spinal SAH [3]. His assertion was that many previous descriptions of spinal SAH were dubious because they were derived from autopsy cases in which the presence of both cranial and spinal blood made it difficult to determine the origin of the hemorrhage. Cases of SAH that were convincingly spinal in origin at autopsy were mostly lumbar, having manifested clinically, with sciatic radiculopathy and symptoms of lower extremity nerve root irritation without the le coup de poignard rachidien (“spinal stabbing pain”). In this paper, we review Michon’s original work as well as more recent descriptions and the understanding of spinal SAH.

## Review

At the time he wrote about le coup de poignard rachidien, Paul Michon was chief of the medical clinic at the Faculty of Medicine of Nancy, France [3]. A translation of his obituary published in La Presse Medicale in September of 1964 revealed that Michon was a well-respected internist in Nancy who served as a corresponding member of the Medical Society of Hospitals of Paris as well as a corresponding member of the National Academy of Medicine [4]. He possessed numerous military decorations, including Chevalier of the Legion of Honor and holder of the Croix de Guerre. He was the director of both a medical clinic and a transfusion resuscitation center in Nancy, channeling a great deal of effort into the study of hematology and perfecting transfusion techniques. He was known for the variety and broadness of his expertise, particularly his research in the fields of dermatology, cardiology, pulmonology, nephrology, endocrinology, and epidemiology. Michon was perhaps best-known though for his work in neurology, for which he had a particular fondness. In addition to his work regarding spinal meningeal hemorrhage, Michon also wrote on topics related to Parkinson’s disease, spinal reflexes, Guillain-Barré syndrome, infectious myelitis, pharyngoesophageal manifestations of botulism, dysglobulinemic neuropathies, and azotemic psychotic encephalitis.

In Michon’s original 1928 French article published in La Presse Medicale, he distinguished spinal SAH of the cervical and thoracic spine from those of the spine containing the cauda equina. He pointed out that it is the cervical and thoracic forms of spinal SAH that he collectively referred to as the “upper” forms, which were the most concerning clinically. While the characteristic interscapular stabbing pain can occur in both, it particularly applies to hemorrhage in the interscapular region of the spinal cord, referred to by Michon as the “dorsal” or “intermediate” form of spinal SAH. Emphasis was placed on the spontaneity and paroxysmal nature of the presentation of the upper forms of spinal SAH, with an overwhelming predominance of spinal symptoms and little, if any, cerebral symptoms. If cerebral symptoms are present, they are non-specific and follow the pattern for any increase in cerebrospinal fluid (CSF) pressure. Although the intense stabbing pain often overshadows other symptoms, Michon additionally described a meningismus-like presentation of neck stiffness, head deflection, exaggerated tendon reflexes, signs of pyramidal tract irritation, and positive Lasegue’s, Brudzinski’s, and Kernig’s signs. SAH of the cauda equina, or “lower form,” is usually accompanied by prominent lower extremity symptoms. He stated that lumbar puncture provides an immediate reduction in symptoms in a cranial to caudal fashion. Michon even suggested that amelioration of symptoms with lumbar puncture should be part of the criteria for diagnosing spinal SAH.

Michon described two patients with presumed spinal SAH who benefitted from a therapeutic lumbar puncture. He detailed their presentation and progression until discharge. Michon also explained how he was certain that his two cases involved spinal subarachnoid hemorrhage as opposed to other etiologies. He excluded the possibility of subarachnoid “effusion,” pointing out the lack of clinical signs of cerebral hemorrhage. Furthermore, he argued that his patients’ symptoms were more consistent with transient spinal cord dysfunction than a destructive intramedullary lesion, further supported by the rapid relief of symptoms following lumbar puncture. Although many of the symptoms would include meningitis in the differential for spinal subarachnoid hemorrhage, Michon argued that the lack of overt signs of infection and the presence of the dramatic stabbing spinal pain more strongly supported the diagnosis of spinal SAH.

Case 1

On January 26, 1925, a 29-year-old male presented with a three-day history of severe interscapular pain, neck stiffness, and pain along his vertebral column, with associated symptoms of vomiting and an intense headache. Earlier the previous month, he had begun experiencing interscapular discomfort and at the beginning of January, he experienced an episode of severe lower back pain following exertion, which required three weeks of rest, two of which he was bedridden. Three days prior to admission, he returned to work, and while walking there, he was overcome by an intense interscapular pain that led him to return home. He was confined to bed, as his symptoms developed over the ensuing three days, and after being admitted, he would shout out in pain with any slight movement.

On examination, the patient was noted to have slight opisthotonus, marked vertebral stiffness, positive Kernig’s and Brudzinski’s signs, and an inability to extend the lower extremities. Tendon reflexes were hyperreactive in all four extremities, with bilateral ankle clonus, having five to six beats. The patient showed generalized hyperesthesia to touch and prick and reported a backache, severe headache, pain and pressure in the eyeballs, and marked photophobia. He additionally reported a three-day history of constipation and forceful emesis. His past medical history was significant for emphysema and chronic bronchitis, attributable to the inhalation of poisonous gas during the war.

Lumbar puncture was performed and yielded 15 cc of a uniformly bloody CSF (the nature of the drainage did not change throughout the procedure). The patient showed an immediate reduction in symptoms, but within seven hours, he returned to his original state with the exception of emesis. He soon thereafter developed a fever of 38°C. A second lumbar puncture was performed and again yielded 15 cc of bloody CSF and, afterward, the patient improved remarkably. He once again became symptomatic several hours later, but much less so than following the first lumbar puncture-he had a discrete headache, mild back stiffness and pain, and he was now able to extend his leg.

About a week later, the patient continued to have a fever of no greater than 38°C, and on February 1, he developed some of his original symptoms. On examination the following day, the patient complained of persistent interscapular pain and neck stiffness. The patient was discharged on February 10 with neck stiffness, a mildly positive Kernig’s sign, brisk tendon reflexes in the lower extremities, and an intense but much more diffuse interscapular pain and headache. He returned for follow-up a week later and appeared to be in good health. On physical examination, the patient was noted to possess only the remnants of a Kernig’s sign.

Case 2

On October 10, 1927, a 17-year-old female with aortic insufficiency, “an irritable, unstable personality,” and a three-day history of constipation presented with a severe stabbing interscapular pain. She also suffered from recurrent epistaxis and large ecchymoses were noted on the patient’s lower extremities, which her family mentioned occurred quite often. She likely suffered from some form of bleeding dyscrasia. She suddenly began experiencing excruciating pain between her shoulder blades, causing her to scream. Her family laid her down, after which she began dry heaving and yelling at the slightest movement. These attacks would occur in intervals of 10 minutes or less.

On initial examination, the patient’s head was turned to the right. She did not respond to questions but followed a command to open her mouth. Every five minutes, the patient would spontaneously let out two or three long screams (which Michon described as being suggestive of internal hydrocephalus). Associated signs included bladder incontinence, paraparesis, and symmetrical signs of pyramidal dysfunction in the lower extremities with exaggerated tendon reflexes, clonus at the ankle, and a positive Babinski sign. There was no change in symptoms by the following day, and due to her inability to tolerate vertebral mobilization, a lumbar puncture was performed under general anesthesia. The procedure found CSF under raised pressure, which was uniformly bloody throughout the procedure. Despite removing 30 cc, the CSF continued to flow briskly but at this point, the procedure was terminated.

On the day after lumbar puncture, a marked improvement in the patient’s symptoms was evident. Her cries of pain were less frequent, and she regained some of her intellectual faculties. She had her first bowel movement since her symptoms began. On examination, two new ecchymoses were noted on her lower extremities. Tendon reflexes were brisk, and a positive Babinski’s sign was found bilaterally. The following day, the patient no longer cried out on movement and became aware of and responsive to questions. She was able to start a liquid diet and began urinating frequently. Her temperature was 37°C during the day and 38°C that night, and she had fever on and off over the next few days. Two days later (October 15), the patient reported a diffuse but less intense headache and painful neck stiffness. Physical examination demonstrated a sluggish pupillary light reflex bilaterally and a positive Kernig’s sign. The Babinski’s sign had disappeared, and urinary continence returned.

The patient continued to improve over the next 10 days but began to decline again on October 25 with weight loss, extreme weakness, posterior headache, limitation of neck flexion, a positive Kernig’s sign, sciatic pain, and continued sluggish pupillary reflexes. The patient also began having urinary incontinence, but Michon argued that this could have been psychiatrically induced due to its irregularity and disappearance when certain individuals were around.

Approximately two weeks later (November 10), the patient continued to exhibit remnants of meningeal irritation-a slightly positive Kernig’s sign and mild pain when pressure was applied over the sciatic nerve. She had to be lifted in order to stand due to generalized weakness. Her slow recovery was attributed to the severity of her heart disease and inability to begin a normal diet. The patient was discharged at the end of November, and Michon mentioned that she had a flare-up of rheumatoid arthritis the following month.

Michon’s view versus atypical presentations

Michon repeatedly made the assertion that le coup de poignard rachidien was part of the criteria for diagnosing spinal SAH, particularly in the interscapular region. Furthermore, he strongly downplayed the importance of cerebral symptoms and made no mention of any other than non-specific signs of increased CSF pressure. Such claims have not gone undisputed. In 1956, Henson and Croft pointed out that sudden severe back pain also occurs in cases of intervertebral disc herniation and other vertebral pathology, and that even with a lumbar puncture, it may be difficult to distinguish mild cases of hemorrhage from these other possibilities [5]. They also noted that the intracranial extension of spinal SAH may quickly cause intracranial symptoms, possibly even occurring before spinal symptoms in cases of severe hemorrhage that occur in the upper regions [5]. They described mental status changes, nystagmus, cranial nerve palsies, and ataxia in a patient with no detectable intracranial pathology, and they attributed these symptoms to increased intracranial pressure (ICP) and intracranial extension of the hemorrhage [5].

In 1975, Caroscio et al. described a case of spinal SAH secondary to a spinal AVM and aneurysm in which the patient had severe spinal pain but also presented with diplopia, incoherent speech, and an unsteady gait-symptoms that proved to be clearly intracranial in origin despite the complete lack of intracranial pathology [6]. The authors of this study also reviewed 50 cases of spinal SAH from the literature and identified intracranial symptoms in 40 of them, predominantly consisting of headaches and including mental status changes, loss of consciousness, papilledema, a decrease in vision, nystagmus, diplopia, seizures, and abducens and oculomotor nerve paresis [6].

In 1988, Barton described a unique case in which a patient presented with a chief complaint of severe sharp chest pain that radiated posteriorly to the back and neck and was exacerbated by breathing and pressure over the sternum [2]. A cardiac workup was negative and she was diagnosed with musculoskeletal pain syndrome [2]. She was discharged but returned symptomatically where a lumbar puncture subsequently revealed SAH. Of note, the patient had no neurological complaints or symptoms in her presentation with the exception of neck stiffness [2].

These examples demonstrate that the presentation of spinal SAH can be much more complex than Michon described and using the stabbing spinal pain in the absence of significant intracranial symptoms, the criteria for diagnosis would likely lead to a gross underestimation of its incidence. This, of course, would have unfortunate consequences, delaying a highly effective therapeutic lumbar puncture. Luckily, the imaging modalities available today have eliminated the need for diagnosis based on symptoms alone.

## Conclusions

Spinal subarachnoid hemorrhage is a rare disease entity. French physician Paul Michon supplied one of the earliest in-depth works regarding this condition in his manuscript titled “le coup de poignard rachidien,” reflecting the intense, “stabbing” spinal pain he believed was most integral to the clinical presentation. Michon conveys this understanding through two detailed case reports. Subsequent authors on this topic have emphasized the possibility of other less typical presentations that can make the clinical diagnosis of spinal subarachnoid hemorrhage even more challenging.

## References

[1] Kim YH, Cho KT, Chung CK, Kim HJ (2004). Idiopathic spontaneous spinal subarachnoid hemorrhage. Spinal Cord.

[2] Barton CW (1988). Subarachnoid hemorrhage presenting as acute chest pain: a variant of le coup de poignard. Ann Emerg Med.

[3] Michon P (1928). Le coup de poignard rachidien, symptôme initial de certaines hémorragies sousarachnoïdiennes [Article in French]. La Presse Medicale.

[4] Larcan A (1964). Paul Michon (1897-1964) [Article in French]. Presse Med.

[5] Henson RA, Croft PB (1956). Spontaneous spinal subarachnoid haemorrhage. Q J Med.

[6] Caroscio JT, Brannan T, Budabin M, Huang YP, Yahr MD (1980). Subarachnoid hemorrhage secondary to spinal arteriovenous malformation and aneurysm. Report of a case and review of the literature. Arch Neurol.

